# Contagious Yawning in African Elephants *(Loxodonta africana):* Responses to Other Elephants and Familiar Humans

**DOI:** 10.3389/fvets.2020.00252

**Published:** 2020-05-08

**Authors:** Zoë T. Rossman, Clare Padfield, Debbie Young, Benjamin L. Hart, Lynette A. Hart

**Affiliations:** ^1^Department of Evolution and Ecology, University of California, Davis, Davis, CA, United States; ^2^African Elephant Research Unit Knysna Elephant Park, Western Cape, South Africa; ^3^Department of Anatomy, Physiology and Cell Biology, School of Veterinary Medicine, University of California, Davis, Davis, CA, United States; ^4^Department of Population Health and Reproduction, School of Veterinary Medicine, University of California, Davis, Davis, CA, United States

**Keywords:** African elephants, *Loxodonta africana*, yawning, contagious yawning, interspecific, intraspecific

## Abstract

While spontaneous yawning is common across all vertebrate classes, contagious yawning is less common and has been observed only in a few species of social animals. Interspecific contagious yawning in response to yawning by humans has been observed only by chimpanzees and dogs. After confirming additional occurrences of intraspecific contagious yawning in a group of captive African elephants previously studied, we further investigated the potential for the same group of elephants to engage in interspecific contagious yawning with familiar human handlers. Ten captive African elephants, most of whom had been previously studied, were observed over 13 nights for evidence of intraspecific contagious yawning. Seven of these elephants were also involved in trials where familiar handlers performed staged yawns, as well as trials with staged non-yawning gapes, or trials with no yawns or gapes. Incorporating previously collected contagious yawning data, we describe nine instances of intraspecific contagious yawning in the elephants. Three of the seven elephants yawned contagiously in response to humans during the interspecific yawning trials. This is the first report of interspecific contagious yawning by elephants in response to yawns by familiar humans.

## Introduction

Spontaneous yawning is an ancestral trait that has been identified in many species across all classes of vertebrates (1). There are several hypotheses relating to the function of yawning, but it is generally accepted that yawning relates to a state change and plays a role in brain activation (1, 2). While spontaneous yawning is an ancestral trait, involving the older parts of the brain, contagious yawning, in which an animal yawns upon seeing another yawn, is thought to be controlled by the neocortex which is essential for complex social interactions (3). Contagious yawning with conspecifics (intraspecific contagious yawning) has been reported in several species of social animals including humans, chimpanzees, baboons, wolves, sheep, and budgerigars (4–9). These studies generally show a positive relationship between familiarity among the participants and likelihood of yawning.

Contagious yawning between members of different species (interspecific contagious yawning) has been observed between humans and chimpanzees as well as humans and dogs (10–13). Interestingly, yawn contagion has been reported in different situations between humans and dogs, but not between dogs. In the studies of contagious yawning with humans in both dogs and chimpanzees, there was no evident difference in yawning responses to familiar versus unfamiliar humans; this was also the case in a study looking at contagious yawning by chimpanzees in response to an android (14).

Contagious yawning is, by nature, a social behavior, but it is not entirely clear what communicative function it serves. Several investigators associate the phenomenon with empathy (4–8). Other investigators have questioned the link to empathy (15). In humans, a contagious yawn can be prompted not only by seeing a yawn, but also by hearing or even thinking about a yawn (16, 17).

Since African elephants have a highly developed cognitive brain (18, 19), and are a social mammal adapted for group living, it is reasonable to expect that they would exhibit contagious yawning with other familiar elephants. Our previous study investigated yawning behavior in captive African elephants at the same study site as the present study, reporting 6 instances of contagious yawning (20), 4 of which were by standing elephants.

As background information for the frequency of yawning by standing elephants at night, in the previous study, we accumulated 73.3 h of observations in the nighttime enclosure; this excluded all 2-min periods that were associated with an elephant arousing. During these non-arousing time periods, we observed only 4 spontaneous yawns by the standing elephants, meaning that the rate of a standing elephant spontaneously yawning apart from the 2-min periods of an arousing elephant that might be yawning was 0.055 yawns/h (4 yawns/73.3 h), or one yawn in over 18 h. Spontaneous yawns by standing elephants outside of arousal episodes within the group thus were extremely rare. This previous study, along with information about elephant-initiated interactions with people at the same study site (21), provided the context for the current study.

One goal of the present study was to confirm and expand upon our previous descriptions of intraspecific contagious yawning among the same group of elephants, at a different time and with some changes in the group composition. The second goal was to determine if the human handlers that regularly interacted with the elephants during the day would evoke yawning from the elephants by yawning in a manner similar to that used in the studies of humans evoking yawns from dogs and captive chimpanzees. These observations were conducted in the early morning, outside the nighttime enclosure, a time when spontaneous yawning would be highly unlikely.

The setting of this study included videorecording of the behavior of the elephants freely moving at night; these recordings were available for perusal later by an investigator. The structured interactions of the handlers with the elephants each morning were a daily routine. As noted, the occurrence of contagious yawning is rare compared with spontaneous yawning, so the number of elephants exhibiting intraspecific and/or interspecific contagious yawns by elephants was expected to be small. With elephants, where opportunities to record some behaviors is limited, but the behaviors are meaningful in understanding elephants' comparative behavior, a documented occurrence in just a few individuals is important. The parameters used in designating a yawn as contagious are presented in detail. Recent examples of reports of just one or two instances of elephant behavior, where details are given, are of sleeping bouts in two wild African elephants in nature (22) and self-identification in a mirror by one elephant (23).

## Materials and Methods

### Ethics

The UC Davis Institutional Animal Care and Use Committee approved this study (Protocol #19968). The UC Davis Institutional Review Board found this study exempt from review (Protocol #1066413-1). The trials with human handlers were integrated into a regular early morning handling procedure because this is soon after the arousal time for the elephants and the trials were part of their daily routine. Participation of the handlers in the study was voluntary, and handlers were informed that they could withdraw their participation at any time with no repercussions. Permissions to conduct the experiment were granted by the Knysna Elephant Park management, and all aspects of the study were overseen by the on-site African Elephant Research Unit.

### Subjects

Data were collected at Knysna Elephant Park, Western Cape, South Africa, from July to November 2017, on ten captive African elephants (seven adult females, one sub-adult female, and two sub-adult males), ranging in age from 9 to 27 years. All ten elephants were part of the intraspecific yawning observations, and seven of the elephants were part of the interspecific trials. These seven elephants were part of a “hands-on” herd that interacted daily with the handlers and tourists. The elephants maintained a cohesive herd with a well-defined social hierarchy. Only two of these seven individuals are related (mother and daughter). The remaining three elephants not involved in the interspecific trials (adult females) were new to the park and did not yet take part in the daily routines of the other elephants with handlers and tourists.

### Intraspecific Yawning Observations

Observations over 13 nights took place between 6:00 p.m. and 6:30 a.m. in July 2017. All 10 elephants were kept in an indoor-outdoor enclosure at night where the indoor section, measured 14 m x 14 m, and was illuminated by heat lamps at night. Two Hikvision low-light cameras (model DS-2CE1582P-VFIR3) with a DVR system that had motion detection (model DS-7116HWI-SL) continuously recorded the elephants in the area. Seven of the elephants could move freely between the indoor and outdoor sections (2 ha) of the enclosure, while three elephants were kept in smaller enclosures (6 m x 4 m) within the main indoor area, for management reasons unrelated to this research. We reviewed the video footage with Hikvision iVMS-4200 software, scoring instances of intraspecific contagious yawning.

Results from the previous study (20) revealed when yawns were most likely to occur in this group of elephants, so observations on intraspecific contagious yawning were focused on final recumbent bouts of the night (generally between 3:00 a.m. and 6:30 a.m.) and the 2 min just after arousal by each individual elephant. We could then observe whether the yawn triggered a contagious yawn from any of the other elephants. Consistent with the previous study, for a yawn to be classified as contagious, the second elephant had to have a direct line of sight to the first elephant and the contagious yawn had to occur no more than two minutes after the end of yawning of the arousing elephant. We made a distinction between contagious yawning by a *standing* elephant in response to a yawn from an arousing elephant, and yawning in an *arousing* elephant in apparent response to a yawn from another arousing elephant. Due to the prevalence of yawning associated with arousal, we cannot be certain that the latter is not two arousing elephants spontaneously yawning at around the same time. Control observations from the previous study showed that spontaneous yawns were unlikely to occur outside of the two-minute period following arousal from a recumbency. As described in our previous study (20), a yawn is characterized as a slow opening of the jaw, a brief frozen open posture and a quick closure of the jaw opening. This behavior is seen most clearly in the attached video clip (Supplementary Video 2) of the elephant yawning in response to a handler yawning.

### Interspecific Yawning Trials

Between July and November 2017, we conducted the trials testing for contagious yawning between handlers and the elephants. We tested seven elephants in this phase with two to three trials a week, dependent on handler availability. Randomized trials took place during the scheduled morning training period, when the handlers worked directly with the elephants. Handlers often introduce new commands or forms of enrichment to the elephants during this period of the day and our trials were structured so that they would integrate as seamlessly as possible with the elephants' daily routine. Due to evidence of bonds between the elephants and their handlers (21), it seemed that yawning between the elephants and the handlers could be influenced by the elephant's familiarity with the handler but in this instance all of the handlers who participated in this study had worked with these elephants for several years, and were very familiar to all the elephants. Two trials were conducted on different elephants at the same time, but the trials were situated such that the two focal elephants in the trials were never adjacent to ensure that trials of one focal elephant did not influence trials of the other focal elephant.

During each trial, the focal handler walked around the elephant (consistent with daily handling) and when he was within eyesight of the focal elephant, he yawned. These staged yawns were intended to be as realistic as possible. The handler was asked to yawn repeatedly, about 10 times, at 1-min intervals, depending on eye contact of the elephant. The number of yawns and the trial length were relatively consistent with similar studies on the chimpanzee contagious yawning (10) and the human-dog contagious yawning (11–13) trials.

We conducted two other types of trials in addition to the yawning trials. Although these elephants had never been taught to mimic, we conducted “gape” control trials to control for yawn mimicry. In these trials the handler walked around the elephant, but instead of yawning, the handler exhibited a short gape. Gapes differed from yawns in that they were shorter in duration, with a smaller opening of the mouth. Gapes also differed from yawns in that they consisted of a smooth opening and closing of the mouth, versus the slow opening and quick snap-shut characteristic of a yawn. There were also control trials with no yawning or gaping where the handler walked around the elephant, as typical during a 10-min morning session, and did not exhibit any yawning or gaping. Four yawning trials, two non-yawn gape trials, and two control trials were conducted for each of the seven elephants. Since any type of trial took time away from normal morning training, we were limited in the number of trials we could run. We sought to maximize the number of yawning trials while still conducting the control trials we found to be necessary.

All trials were recorded by video cameras hand-held by a recorder, for subsequent analysis. The elephants and the handlers were recorded, when possible, from a side-view perspective from 10-20 m away so as not to interfere with the trial. During yawn and gape trials, handlers were asked to make a fist each time they performed a yawn or gape so that a correct count of behaviors could be obtained if the handler was not facing the camera, or was partially obscured by the elephant. The same investigator (ZR) reviewed all videos using VLC Media Player software.

### Statistical Evaluation

For intraspecific contagious yawning, the goal was to evaluate the incidence of a standing elephant in the enclosure at night spontaneously yawning within 2 min of a yawn by an arousing elephant. As mentioned above, the rate of yawning by standing elephants, apart from arousal events, is extremely low (1 yawn in 18 h). For interspecific yawning, the goal was to assess whether trials with staged yawns by a handler would provoke a response of a contagious yawn by the elephant; trials with gapes and no special behavior by the handler were conducted for comparison.

## Results

### Intraspecific Contagious Yawning

During the 13 nights with recorded videos, 17 spontaneous yawns were observed in association with arousal from a recumbency. Three yawns meeting our specific criteria for contagious yawns were observed from standing elephants. Two of these yawns were in response to the yawns of another elephant just after she had arisen from a recumbency, and one occurred 40 s after the yawn of another standing elephant (Madiwa and Mashudu). A video of the Madiwa/Mashudu yawn sequence is available in supplemental materials (Supplementary Video 1). This contagious yawn was unusual because standing elephants rarely yawn aside from arousal from a recumbency. In Table 1, details of the three yawns meeting our specific criteria for contagious yawns (Table 1A) from the present study are listed along with the six contagious yawns from the previous study (Table 1B). We chose to include intraspecific contagious yawns from the previous study as this study site uniquely and specifically allows for monitoring contagious yawning. Including all representative data from this group of elephants provides a fuller picture of the contexts for contagious yawning, given our small sample size. As mentioned in the above section the chance of a spontaneous yawn occurring within the 2-min period of the yawn of an arousing elephant yawning is < 0.001.

**Table 1A:** Intraspecific contagious yawning episodes.

**Initiating elephant**	**Responding elephant**	**Description**
Madiwa	Mashudu	One adult female (Madiwa) and one sub-adult male (Mashudu). Mashudu had been standing and had no recumbent bouts that night. Madiwa, who was also standing and had no recumbent bouts, begins to yawn. Mashudu then starts yawning 34 s later.
Thato	Shungu	One sub-adult female (Thato) and one sub-adult male (Shungu). Shungu had been standing and had no recumbent bouts that night. Thato, initially lying down, starts standing and then yawns. Shungu then starts yawning 4 s later. Occurred in association with the final recumbency for Thato.
Thato	Madiwa	One sub-adult female (Thato) and one adult female (Madiwa). Madiwa had been standing and had no recumbent bouts that night. Thato, initially lying down, starts standing and then yawns. Madiwa then starts yawning 34 s later. Occurred in association with the final recumbency for Thato.

**Table 1B:** Previously published details of postulated intraspecific contagious yawning episodes (19).

**Initiating elephant**	**Responding elephant**	**Description**
**Contagious yawning in a standing elephant**		
Mashudu	Nandi	One sub-adult male (Mashudu) and one adult female (Nandi). Nandi had been standing for over 5 min. Mashudu, initially lying down, starts standing and then yawns while starting to stand. Nandi then starts yawning 18 s later with no overlap in yawning times. Occurred in association with the final recumbency for Mashudu.
**Contagious yawning in arousing elephants**		
Mashudu	Keisha	One sub-adult male (Mashudu) and one adult female (Keisha). Both elephants initially lying down. Mashudu stands first, and then Keisha starts standing. Mashudu starts yawning, and Keisha starts yawning 2 s later with a 2 s overlap in yawning times. Occurred on final recumbency for both elephants.
Nandi	Thandi	Two adult females (Nandi and Thandi). Both elephants initially lying down, then both start standing. Nandi starts yawning, and Thandi then starts yawning 5 s later, with an 8 s overlap. Occurred on the final recumbency for both elephants.
Nandi	Shungu	One adult female (Nandi) and one sub-adult male (Shungu). Both elephants initially lying down, then both start standing. Nandi starts yawning, and Shungu then starts yawning 6 s later with a 3 s overlap. Occurred on the final recumbency for both elephants.
Keisha	Thato	One adult female (Keisha) and one sub-adult female (Thato). Both elephants initially lying down, then both start standing. Keisha starts yawning, and then Thato starts yawning 16 s later, with no overlap in yawning times. Occurred on the final recumbency for both elephants.
Mashudu	Clyde	One sub-adult male (Mashudu) and one adult male (Clyde). Both elephants initially lying down, then both start standing. Mashudu yawns, and then Clyde yawns. Information on delay between the starting of yawning and overlap times could not be discerned. Occurred on the final recumbency for both elephants.

### Interspecific Contagious Yawning

The handlers yawned (staged yawns) a mean of 8.8 times per trial (SD = 2.7), with a mean trial duration of 10.4 min. There was an average of 10.6 gapes by handlers per trial (SD = 1.8), with a mean trial duration of 10.3 min. The mean duration of the control trials was 10.2 min.

During her initial trial, one elephant, Keisha, yawned contagiously in response to the handler's yawns. However, at the end of the trial, the handler responded in a positive way, praising the elephant repeatedly (Supplementary Video 2). Keisha then yawned five more times during her remaining trials, including once during a yawning trial, twice during gape trials and twice during control trials. She also yawned four times during the trials of adjacent elephants, including a yawning trial, two gape trials and a control trial.

Following this initial trial with Keisha, we reminded the handlers to not respond to an elephant's yawn in any way that could potentially reinforce or encourage (or discourage) yawning behavior. We included the results from Keisha's first yawning trial in the data analysis since her initial contagious yawn was performed prior to any behavioral reinforcement, but excluded the results from all her subsequent yawning, gape, and control trials in data analyses.

**Figure 1:**
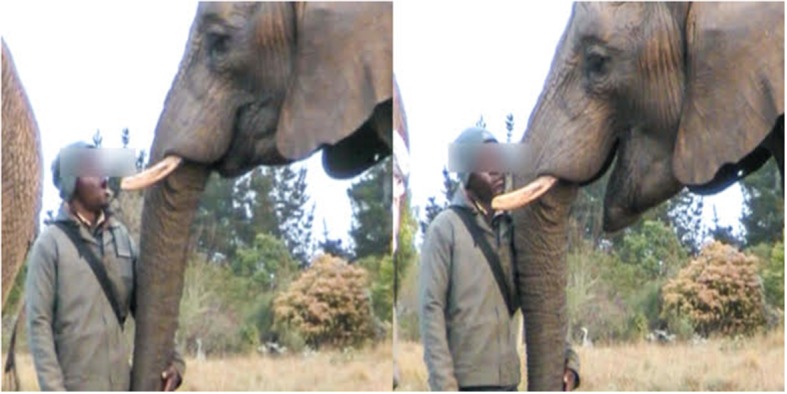
Contagious yawn sequence. The handler yawns in the left panel, quickly followed by a contagious yawn from the focal elephant in the right panel. A video of this sequence is available in supplemental materials (Supplementary Video 3).

**Figure 2:**
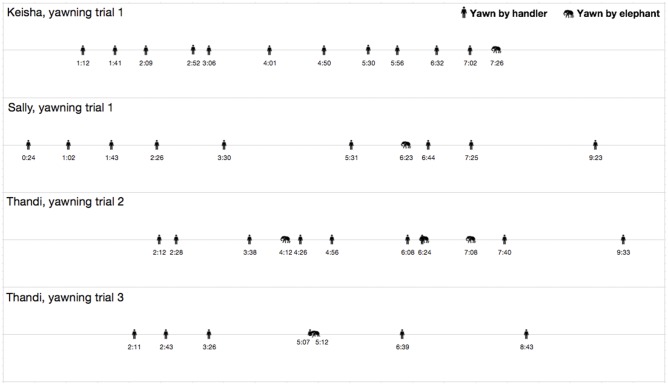
Contagious yawning trial timelines. Timelines of each of the four trials that resulted in a contagious yawn from the focal elephant, showing the sequence of staged yawns performed by the handler and the yawn(s) in response from the focal elephant.

Across the 24 yawning trials of the six elephants, excluding Keisha, five contagious yawns were observed in three trials from two of the six elephants (Figure 1). Across the 12 gape and 12 control trials of the six elephants, one yawn was observed during a gape trial and one during a control trial. When the first contagious yawn of Keisha is included, a total of six contagious yawns were observed from three elephants in four separate trials (Figure 2). In trials where contagious yawning occurred, the handler yawned a minimum of 3 and a mean of 6.3 times before the elephant yawned contagiously (Figure 1). In these trials, a minimum of 2 min and a mean of 4.5 min elapsed between the initial handler yawn and the elephant's contagious yawn. The mean duration of the contagious yawns from the elephants was 6.8 s, which is consistent with the duration of spontaneous yawning observed in this group of elephants.

## Discussion

### Intraspecific Contagious Yawning

The results from our nighttime observations, with three instances of contagious yawning meeting the specific criteria, expand upon and confirm our previous report of intraspecific contagious yawning between elephants (20). There were two instances where a standing elephant yawned in response to an arousing elephant yawning and one from a standing elephant yawning in response to another standing elephant yawning. This latter instance expands the contexts in which contagious yawning in African elephants may be expected to occur. The most frequent context is that where a standing elephant yawns in response to yawning by an elephant arousing or just after arousing and standing. The next most frequent context is an arousing elephant yawning in response to yawning by another arousing elephant. This was not seen in this present study, but was seen in the previous study. The third context is a standing elephant yawning in response to yawning by another standing elephant. To address the issue of a spontaneous yawn occurring in a standing elephant being mistaken for a contagious yawn, we calculated the probability of a spontaneous yawn by a standing elephant, being < 0.001. Table 1 outlines the nine occurrences of intraspecific contagious yawns seen in the two studies. The varieties of contexts are mindful of the different contexts of contagious yawning in humans, ranging from directly looking at another person, to just hearing someone talking about yawning (4, 16).

### Interspecific Contagious Yawning

Trials to test for interspecific yawning were conducted early in the morning when the elephants had just been taken from the nighttime enclosure. This was a few hours after the time when spontaneous yawning and contagious yawning between elephants had been observed. As noted in the introduction, spontaneous yawning was almost always seen in association with arousal from recumbent sleeping or resting bouts, and not at other times. Thus, yawns that occurred during these handler trials are unlikely to have been spontaneous.

The testing protocol followed was similar to that used in trials with dogs and chimpanzees to observe for yawning by the animal in response to yawning by the human that was facing the animal. In trials on dogs and chimpanzees, the human yawned several times before a yawn occurred by the animal, if it occurred. The durations and patterns of contagious yawning in handler trials were typical of spontaneous yawns and of those seen in the contagious yawns between elephants. In contrast with prior studies investigating interspecific contagious yawning, our study was conducted using only familiar humans. We do not know if elephants would respond contagiously to the yawn of a strange human.

The observations of Keisha during yawning trials, in which she initially yawned in response to the handler's yawn and then yawned several times during her control and gape trials, and those of other elephants, provide evidence for her yawns being provoked in ways other than directly seeing a yawn. Given that spontaneous yawning is infrequent during periods of time not associated with arousal, it is unlikely that Keisha's prevalent yawning was occurring as spontaneous yawns. This yawning behavior by Keisha seems analogous to observations on human yawning being triggered by thinking about a yawn (17), suggesting that Keisha began to associate the trial context with handlers yawning. An alternative explanation is that the yawning was a reflection of the positive reinforcement from the handler after her initial contagious yawn, implying that she then began yawning deliberately due to the positive association of yawning with praise. Both possibilities illustrate the involvement of the neocortex in contagious yawning (discussed in the introduction), as opposed to spontaneous yawning.

Whether yawning, let alone contagious yawning, occurs in wild free-ranging African elephants has not been investigated at this point. The study of two matriarchs in Botswana from two herds of free-ranging wild elephants, using a trunk-movement proxy for sleep, found that the elephants slept about 2 h per night, standing or in recumbency (22). There was no video recording of the elephants, so it is unknown whether or not contagious yawning, or even spontaneous yawning, may have occurred. Based on our studies, elephant yawns are rather subtle. mostly occurring at night, and would likely be missed by observers of wild elephants.

Including the first interspecific yawn of Keisha, we documented six interspecific yawns in three elephants. While the number of yawns is small, the observations meeting specific criteria should be sufficient to establish that elephants can, and do, display yawning in response to seeing a familiar human yawning. As mentioned above, at least two other notable behaviors have been recently documented in elephants based on just one or two individuals: nighttime sleeping (22) and self-recognition in a mirror (23).

A possible function of contagious yawning among elephants living in nature, assuming it occurs, and based on the presumed function of yawning, is brain arousal and activation. Yawning by an arousing elephant, leading to a contagious yawn from elephants already awake, or another simultaneously arousing elephant, could facilitate a state of higher awareness and arousal throughout the herd. Although our data are on captive elephants, these elephants are a well-integrated herd with group dynamics similar to wild elephants where it would be advantageous for an aroused state to quickly spread through a herd in response to an external stimulus that caused the arousal of some individuals.

The findings presented here with regard to contagious yawning among a close group of captive elephants, plus the occurrence of some contagious yawning of the elephants in response to staged human handler yawning, are consistent with the perspective of the highly-developed brain of elephants. Elephants have a larger associative neural cortex than other mammals; a neural cortex that is involved in social-empathic responses (18, 19). As discussed in the introduction, several studies associate contagious yawning with empathic behavior. In elephants, von Economo neurons (VENs) have recently been discovered in the neocortex, and are virtually identical to the VENs of humans and chimpanzees (19, 24). These neurons subserve the mental attributes of empathy and compassion in humans. Although we cannot infer the emotions or behavioral motivations of elephants, the presence of VENs in elephants suggests that elephants and humans share an important mediating substrate for social-empathic behaviors. While there is no currently proven link between contagious yawning and empathy thus far, the potential empathic aspect of contagious yawning in elephants would be consistent with the cytoarchitecture of the elephant brain.

## Data Availability Statement

All datasets generated for this study are included in the article/Supplementary Material.

## Ethics Statement

The studies involving human participants were reviewed and approved by The UC Davis Institutional Review Board found this study exempt from review (Protocol #1066413-1). Written informed consent for participation was not required for this study in accordance with the national legislation and the institutional requirements. The animal study was reviewed and approved by The UC Davis Institutional Animal Care and Use Committee approved this study (Protocol #19968). Permissions to conduct the experiment were granted by the Knysna Elephant Park management, and all aspects of the study were overseen by the on-site African Elephant Research Unit.

## Author Contributions

ZR, LH, BH, CP, and DY conceived and designed study. ZR and CP collected data. ZR reviewed video footage and analyzed data. ZR, LH, BH, and CP drafted and edited manuscript.

## Conflict of Interest

The authors declare that the research was conducted in the absence of any commercial or financial relationships that could be construed as a potential conflict of interest.

## Supplementary Material

Supplementary Video 1 Intraspecific contagious yawn sequence. The elephant on the far right begins to yawn at 0:13. At 0:45, the elephant in the center (previously blocked from view) begins to yawn in response.

Supplementary Video 2 Initial contagious yawn from Keisha. Keisha yawns after a series of handler yawns. The handler pats her and tells her “Good girl, Keisha” in response.

Supplementary Video 3 Interspecific contagious yawn sequence. The handler yawns at 0:02, and the elephant yawns in response at 0:09 during an interspecific yawning trial.
